# Inosine Triphosphate Pyrophosphatase (ITPase): Functions, Mutations, Polymorphisms and Its Impact on Cancer Therapies

**DOI:** 10.3390/cells11030384

**Published:** 2022-01-24

**Authors:** Mazin A. Zamzami

**Affiliations:** 1 Department of Biochemistry, Faculty of Science, King Abdulaziz University, Jeddah 21589, Saudi Arabia; mzamzami@kau.edu.sa; 2 Centre of Artificial Intelligence in Precision Medicines, King Abdulaziz University, Jeddah 21589, Saudi Arabia

**Keywords:** inosine triphosphate pyrophosphatase (ITPase), polymorphism, deamination, cancer therapy, purine metabolism

## Abstract

Inosine triphosphate pyrophosphatase (ITPase) is an enzyme encoded by the *ITPA* gene and functions to prevent the incorporation of noncanonical purine nucleotides into DNA and RNA. Specifically, the ITPase catalyzed the hydrolysis of (deoxy) nucleoside triphosphates ((d) NTPs) into the corresponding nucleoside monophosphate with the concomitant release of pyrophosphate. Recently, thiopurine drug metabolites such as azathioprine have been included in the lists of ITPase substrates. Interestingly, inosine or xanthosine triphosphate (ITP/XTP) and their deoxy analogs, deoxy inosine or xanthosine triphosphate (dITP/dXTP), are products of important biological reactions such as deamination that take place within the cellular compartments. However, the incorporation of ITP/XTP, dITP/dXTP, or the genetic deficiency or polymorphism of the *ITPA* gene have been implicated in many human diseases, including infantile epileptic encephalopathy, early onset of tuberculosis, and the responsiveness of patients to cancer therapy. This review provides an up-to-date report on the ITPase enzyme, including information regarding its discovery, analysis, and cellular localization, its implication in human diseases including cancer, and its therapeutic potential, amongst others.

## 1. Introduction

**Figure 1:**
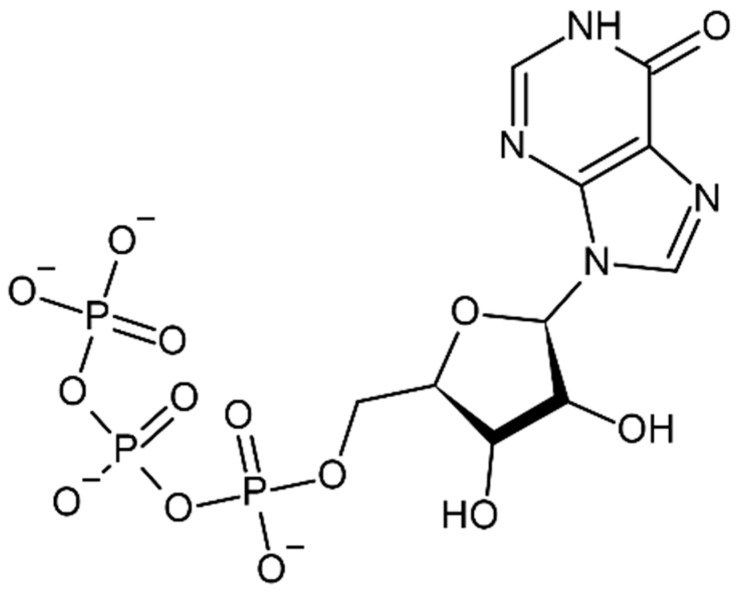
Chemical structure of Inosine triphosphate (ITP).

Inosine triphosphate pyrophosphatase (ITPase) was first discovered in human erythrocytes in 1964 by Liakopoulou and Alivisatos. The tasks of ITPase were different from those of ATPase, competitively inhibited by adenine derivatives and dependent on the presence of magnesium (Mg^2+^). By using partially isolated ITPase, it was shown in the year 1970 by Vanderheide that the enzyme delivers PPi from human erythrocytes [1]. Almost a decade later, it was then distinguished in terms of enzyme kinetics for ITP, isolated 2-3000-fold from human erythrocytes, and studied to understand its work against ATP (Figure 1), GTP, CTP, and UTP. Until the beginning of the century, two major findings came in human *ITPA*. Firstly, in the very same year more attention was paid to the subcellular localization and tissue distribution of *ITPA*. It was found that bone marrow fibroblasts had significantly much higher activity in comparison with general erythrocytes. Secondly, the results from Vanderheiden demonstrate that *ITPA* was highly particular for ITP and dITP and also found that XTP was a substrate [2]. In the year 2001, several studies had been carried out to understand the expression of *ITPA* in *E. coli*. With the help of recombinant technology and northern blots, it was broadly studied in 24 tissues and notably enlisted in the pancreas, liver, heart, testis/ovary, thyroid gland, adrenal gland, etc. Purified recombinant *ITPA* created the accelerator and set the stage for future developments, as well as the identification of further substrates, such as the triphosphates of agent base analogues 6-hydroxyaminopurine (HAP) and 2-amino-6-hydroxyaminopurine [3].

## 2. *ITPA*: A Crucial Metabolic Enzyme

**Figure 2:**
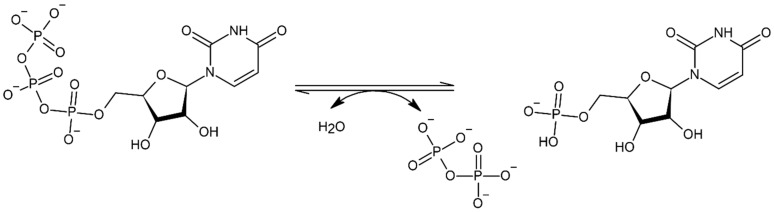
Chemical formation of ITP molecule.

**Figure 3:**
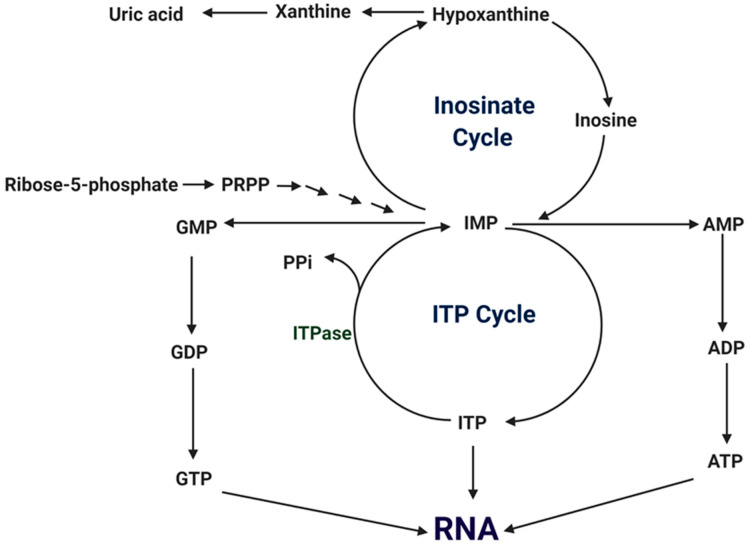
A schematic depiction of ITP cycle.

**Figure 4:**
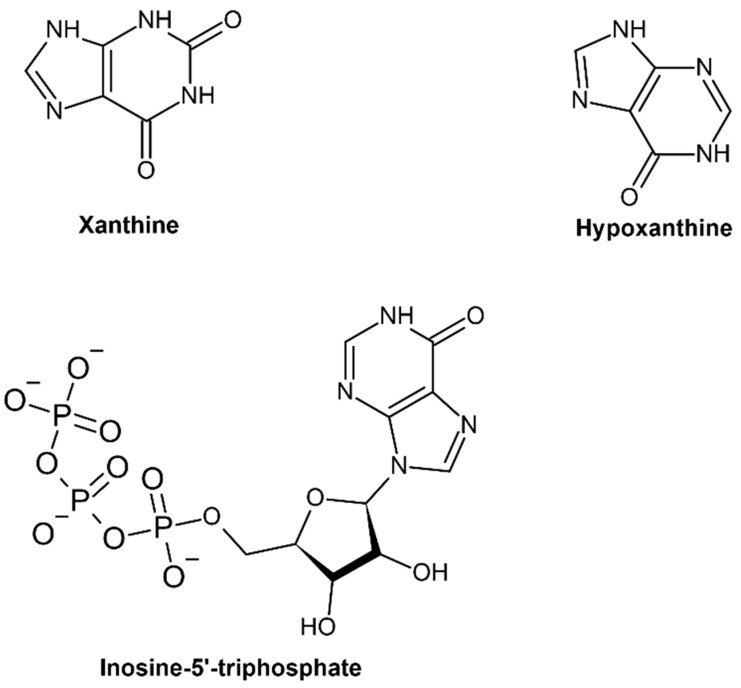
Chemical structure of contributing molecules for the ITP cycle, namely xanthine, hypoxanthine and inosine 5-triphosphate.

Inosine triphosphate pyrophosphatase is a ubiquitous protective key enzyme which regulates the contamination of cells at non-canonical levels. It does not allow the accumulation of nucleotides. The deamination of purine bases gives rise to inosine and xanthine nucleotides, which break down through ITP and PPi (Figure 2). Xanthine nucleotide (XTP) is also a substrate, but not much activity has been seen towards other nucleoside triphosphates, and in IDP or IMP it is completely absent (Figure 3 and Figure 4) [4]. ITPase helps in preventing the storage of ITP and lowers the chance of inosine nucleotides merging into nucleic acids. It was isolated, purified and characterized from human erythrocytes. With the help of a two-step colorimetric assay, the enzymatic properties of ITPase in human erythrocytes have been studied. It was found that in the cytosol portion the ATP and GTP deamination activities were not much enhanced [5]. The activity of *ITPA*s has been studied in different tissues such as bone marrow, skin fibroblasts, lymphoid lines, amniotic fluid, and erythrocytes. Average ITPase activity ranges between 4.9 and 294 units/mg protein. This enzyme has a broad range-specific activity in erythrocytes, from 15 to 722 pmol/h/g Hb [6]. Tested samples of leukocytes showed the lowest ITPase activity. Therefore, a lower presence or the absence of ITPase activity is a discrete characteristic which may be seen in erythrocytes and other types of cells.

Many studies have been carried out to measure the Km value for *ITPA*, and it was found that in every case, the ideal pH is alkaline and the need for divalent ion such as Mg^2+^ or Mn^2+^ is seen. A study of different samples of 6000 individuals revealed that seven samples showed that the deficit in ITPase activity was responsible for the presence of a high level of ITP. The short arm of chromosome 20 represents the gene that codes for ITPase, *ITPA* [7]. However, no genes have been cloned and characterized in mammalian erythrocytes; only a few rat tissues and the liver of rabbit. Structure-based identification with relevant biochemical analysis was revealed from *Methanococcus jannaschii* in a novel bacterial nucleoside triphosphate pyrophosphate, Mj0226. A protein found in yeast, named Ham 1p, has been shown to be the result of a gene controlling the response of yeast strains to 6-*N*-hydroxylaminopurine (HAP). With about 30% sequence identity, this yeast protein is analogous to j0226 protein [8].

The ITPase is an α/β homodimeric protein that contains 194 amino acid residues, which form a 45 kDa dimer [5]. The two globular lobes of the enzyme are supported by a central elongated mixed β sheet. The binding of ITP takes place between the lobes, which are adjacent to the dimer interface. This homodimer contains a catalytic site which is found towards the periphery and a specificity pocket which is deeply buried in the dimerization of each monomer lobe. The particular mechanism has not yet been explained. However, it is believed that Mg^2+^ is needed for the catalytic activity, which takes place via acid–base chemistry. It has been seen that ITPase works best in the presence of reducing agents such as DTT in an alkaline pH. ITPase does not differentiate between ribose and deoxyribose sugar and breaks down phosphoanhydride attachment in non-canonical NTPs and dNTPs with the same interest [5,7].

The protein substrate complex has been elucidated through X-ray crystal structure. It was found that there are some unfolded preserved remains that indicate the control of substrate particularity. Almost 40 years later, the exact mechanism of the ITPase in distinguishing between canonical and noncanonical (d)NTPS has still not been completely revealed. Competitive inhibition has been seen only in human ITPase by inosine 5′-diphosphate; it has not been seen in NMPs or any other trialled nucleoside. A study showed the strong inhibition of enzymes by ions such as Ca^2+^, Cd^2+^, and Co^2+^ [5,7]. The only nucleoside tested was inosine 5′-diphosphate. Competitive inhibition is shown in human ITPase at inosine 5′-diphosphate. However, positive cooperativity is seen only in the *E. coli* ITPase ortholog, RdgB [9]. To date, ITPase biochemistry has been overlooked. However, some advanced studies have been conducted regarding substrate selectivity and clinically relevant variants. ITPase plays a very important role in purine metabolism. However, research gaps exist in understanding the biochemistry and function of ITPase. There are several roles of ITPase which are still unknown, such as the role of ITP in cellular substrate inhibition. It effects the regulation of the concentration of ITP, IPM, or inosine inside the cell, and whether the enzyme is allosterically regulated or post-translationally modified [9].

Substrate inhibition has been studied in different sources, and it was found that when a protein source comes from the whole-genome extract of erythrocyte cells, it is not subjected to substrate inhibition [10]. However, in enzymology, ITPase is defined as a source of protein inhibition. The central idea of this study tells us that the substrate binding of ITPase activity may have a pivotal role in the development of therapeutics.

## 3. *ITPA* Mutations and Association with Clinical Disease

It is believed that mutation significantly affects the structure of a protein. It mostly causes a change in the length of 194 residues of amino acids, and may result in non-functional proteins. Patients with a homozygous mutation as a result of deletion, duplication or frameshift mutation in erythrocyte or fibroblast cells exhibit severe damage to or the complete failure of protein activity [11]. Out of seven clinically relevant *ITPA* variants, one is a duplication, the other a nonsense mutation and a deletion, and the remaining four are single-nucleotide substitutions. Nonsense, duplication and deletion mutations are thought to be against variants and are very infrequent. All three mutations were pointed out in a group of patients with infantile encephalopathy.

In mutants (c.359_366dupTCAGCACC), a duplication mutation of 8 base pairs results in a frameshift mutation at the 123 position of an amino acid, which was speculated to produce 225 amino acid polypeptides with changes in amino acid sequence. A deletion of 1874 base pairs was seen in a mutant (c.264-607_295 + 1267del), which completely spanned exon 5 and resulted in a frame transcript. A premature stop codon at the 151 position of the amino acid is introduced as a result of a nonsense mutation in c.452G > A, which results in the shortening of C-terminal protein, which may tremendously preserve the specificity pocket that contains SHR, a trademark sequence at positions from 176 to 178 [4,11].

Out of the seven nucleotide mutants listed, four are clinically relevant and the most studied variant is the c.94C > A (p.Pro32Thr). Two single-nucleotide mutant/variants affect protein structure in several ways, including reduced catalytic activity and decreased stability and expression of the full-length transcript [11]. This type of point mutation is responsible for mRNA splicing events at exon 2 and 3, which results in a non-functional protein. This event is reduced in wild-type cells, while this increases almost 3-fold in homozygous mutants. Other studies showed that the replacement of proline with threonine at position 32 is responsible for proteins having a reduced stability and lesser catalytic activity [4]. Furthermore, the *ITPA* c.94C > A sequence variant has been shown to be linked with the susceptibility to advanced drug reactions in azathioprine-treated patients. In addition, the *ITPA* c.94C > A allelic variant has been proposed as being responsible for destroying the exonic splicing silencing element in exon 2 in peripheral blood leukocytes patients; ultimately, this led to a change in the structure of the ITPase and contributed to its deficiency (the *ITPA* c.94C > A and c.124 + 21A > C (g.IVS2 + 21A > C) sequence variants led to misplacing of the *ITPA* gene) [12]. In adult hematological malignancy patients, the *ITPA* 94C > A variant has been reported to be linked to a substantial surge in the total heteroplasmic/homoplasmic mutations in the mitochondrial DNA, which implies that a decrease in the activity of the ITPase may likely give rise to changes in the mitochondrial *ITPA* and a possible association with mitochondrial DNA defects [13].

The c.532C > T (p.Arg178Cys) mutant is infrequent and was introduced in patients suffering from infantile encephalopathy. One of the residues that form the SHR signature sequence for ITPase is Arg-178, which is highly conserved in the ITPase. In vivo and in vitro site-directed mutagenesis studies have shown that this position is prime for ITPase activity. Additionally, with the help of many software programs, it is supposed to link with the nucleobase of incoming (d)ITP at two positions, which is thought to result in a non-functional protein. The other two nucleotide mutants do not affect the protein structure [14].

Additionally, it was found that ITPase deficiency is linked to the severity of organ dysfunction. For example, in families affected by *ITPA* mutations involving both alleles, these people expressed muscle heart failure, which was detrimental at an early age. They also possessed features that resembled Martsolf syndrome, a genetic disorder that affects the eye and the brain (ITPase deficiency leads to Martsolf-like syndrome with a lethal infantile dilated cardiomyopathy) [12]. The severity of the *ITPA* loss has been tested in mice. For example, *ITPA* knockout in a mice model leads to the accumulation of inosine nucleotides in both the nucleotide and RNA poll, resulting in the death of the mice at infancy. These mice showed a distinctive feature that resembled growth retardation and the disorganization of the cardiac myofiber (ITPase-deficient mice show growth retardation and die before weaning) [15].

## 4. *ITPA* Mutations and Therapeutic Implications

In the last few years, the pharmacogenetic importance of *ITPA* mutations has been reported. To date, studies suggest that a minimum of 30 percent polymorphism is identified in the *ITPA* gene. Of thirty studies, seven were demonstrated to be clinically suitable (Table 1).

According to these studies, the *ITPA* position influences the results of thiopurine therapy and hepatitis C treatment [11]. *ITPA* mutation is also associated with young-onset tuberculosis susceptibility and is responsible for early infantile encephalopathy. Detailed study of the physical expression of the genes associated with *ITPA* has shown a lot of advancement in clinical treatment [4].

In the year 2009, it was found that the Asian population retains an *ITPA* variation of about 14% to 19% which is the highest among the total 5% of global inhabitants that hold onto c.94C > A (p.Pro32Thr) *ITPA* variation [16]. Subsequently, many other kinds of polymorphisms have been discovered. Clinical mutations are very occasional; in fact, data suggest that the low ITPase activity is responsible for the *ITPA* polymorphism in almost 75% of the affected population [13]. Variation in the genotype may result in a downfall in various levels of ITPase activity. It is crucial to understand that the results prevail from erythrocytes and the ITPase activity measured is different for different cell types, even if it is from similar individuals. Various studies have been carried out to understand the ITPase activity and common types of polymorphism in erythrocytes c.94C > A (p.Pro32Thr) and c.124 + 21A > C (g.IVS2 + 21A > C). A polymorphism in Caucasian populations, the c.94C > A (p.Pro32Thr), shows a serious downfall in ITPase activity and the average activity for heterozygous individuals is ~25 %, whereas homozygous individuals maintain less than 1% of the wild-type levels [14,17]. Moreover, an IPTA genotyping study in a quarter of the Tunisian population showed that polymorphisms in that population had reduced metabolic functioning of the enzyme [18]. It was also found that the Azathioprine-induced neutropoenia in PR3+ microscopic polyangiitis was probably due to an IPTA 94C > A mutation [19].

The c.124 + 21A > C variant results in a small reduction in ITPase activity, and heterozygotes maintain about 60% of the wild-type levels while homozygous individuals maintain about 30 % of the wild-type levels [4]. The resulting c.94C > A (p.Pro32Thr) and c.124 + 21A > C compound heterozygosity demonstrate a major reduction in ITPase activity and allowed them to maintain 8% of the wild-type levels [16]. Previous studies have been conducted in erythrocytes in order to understand the common polymorphisms of ITPase activity, c.94C > A (p.Pro32Thr) and c.124 + 21A > C. The current spectrum of genotype status shows a decrease in ITPase activity [20].

### 4.1. Infantile Encephalopathy

Recently, Kevelam et al. found that one of the reasons behind early infantile encephalopathy is a recessive *ITPA* mutation. With the help of whole-exome sequencing (WES) and magnetic resonance imaging (MRI), researchers came to know of a distinctive pattern of MRI, which shows a recessive mutation of the *ITPA* gene [11]. This study was conducted on seven patients and six patients died before 2.5 years of age. Those patients faced seizures, developmental delay, and very serious progressive microcephaly from birth. Of the different profiling methods, the tests samples showed normal levels of purine and pyrimidine. However, it affected the heart function and resulted in a lower number of RBCs. Homozygous genetic mutation is one of the main reasons behind this, including missense mutations, frameshift mutations, nonsense mutations, and gene deletions. Missense mutations are responsible for the change in the primary structure of the amino acid. In such cases, it is observed that Arg-178 is replaced with cysteine [11,21]. Moreover, a study also showed that ITPase deficiency may lead to refractory epilepsy, microcephaly, and neurodevelopmental disease [22]. The effect of ITPase deficiency on neural epilepsy has been confirmed in knockout mice [23], which is also supported by another mice study that showed ITPase deficiency may lead to growth retardation [24].

### 4.2. Cancer Chemotherapy (Thiopurine Treatment)

**Figure 5:**
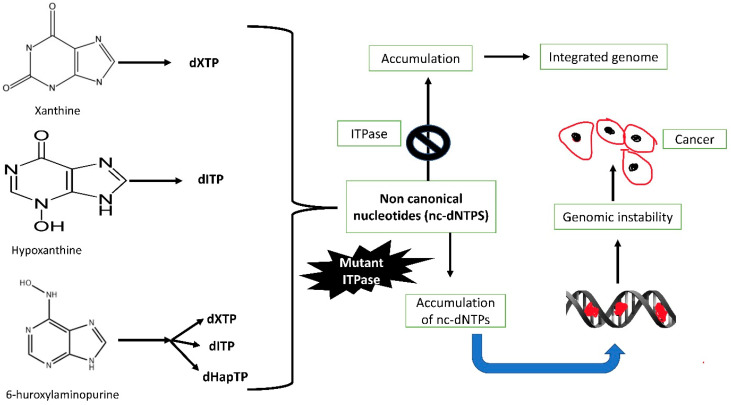
Role of ITPase in genomic stability and cancer.

A different group of studies showed that *ITPA* variation has a direct role in the increased toxicity of thiopurines. It is important to understand that not each and every *ITPA* polymorphism is related to thiopurine toxicity. Nowadays, thiopurines such as azathioprine or 6-mercaptopurine are extensively used to treat several diseases, such as inflammatory bowel diseases, ulcerative colitis, Crohn’s disease, cancer, and organ transplants (29,30). Patients with *ITPA* mutation have many mild to severe side effects, which include rashes, liver toxicity, inflammation of the pancreas, and aplastic anaemia. The presence of thiol-contacting NTPs is responsible for such toxic conditions. These life-threatening side effects sometimes result in either discontinuation or dosage alteration. In order to minimize drug adversity, scientists have suggested the inclusion of pre-screening of the drug for use in patients affected by *ITPA* polymorphism [11,25]. A study also showed that aberrant activity of ITPase caused the accumulation of non-canonical nucleotides that may induce DNA damage and mutagenesis (Figure 5) [26], which was also supported by another study that confirmed ITPA as having a key role in humans to protect DNA [27].

Patients suffering from Crohn’s disease show resistance to 6-mercaptopurine therapy, which is successfully replaced using 6-thioguanine [14]. The main reason behind thiopurine toxicity is still unknown. Some studies do not suggest any correlation between *ITPA* mutation and drug toxicity, and there is still no specific set of rules that have been identified between the *ITPA* variation and drug toxicity. However, potential factors could include race, sample size, or drug reaction. Instead of azathioprine or 6-mercaptopurine, it is encouraged to used 6-thioguanine. In 2014, Matimba et al. demonstrated an experimental method called the “three-tiered” strategy to reduce the failure related to the clinical use of this drug [28].

In addition, it was previously found that the combined treatment of thiopurine with anti-tumour necrosis factor (TNF) can minimize the risk of anti-drug antibody formation that may attenuate the anti-TNF agent’s response. The effectiveness of thiopurine is different according to racial differences. For instances, the effectiveness of thiopurine treatment was found to be significant in East Asian populations, including Korea, China and Japan [29]. The occurrence of leukopenia is lower in Caucasian populations than in Asian populations, and hair loss is exceptional in Caucasians and not abnormal in Japanese patients, while the standard dose of thiopurines in Europe (AZA 2–2.5 mg/kg/day) and in Japan (AZA 1–2 mg/kg/day) differs [15,30]. Additionally, a study also showed that silencing the ITPase gene led to the induction of apoptosis in folate single-wall-nanotube-treated SKBR3 cancer cells [31].

The genetic polymorphism of the enzymes affects the metabolism of thiopurine. A variation in individual thiopurine metabolism is responsible for some of the adverse reactions. Pharmacogenetic studies have been reported in leukaemia, organ transplantation and IBD and some pharmacogenetic predictors have been found and are being used in clinical practice [25,32].

### 4.3. Tuberculosis Treatment

Recently, it was shown that *ITPA* overexpression is implicated in juvenile tuberculosis. By using next-generation sequencing techniques, *ITPA* polymorphism was identified in multiple juvenile patients from different families suffering from tuberculosis, with the expression of g.19176G > A and c.94C > A (p.Pro32Thr) related to juvenile TB patients [4]. Among many variants of the *ITPA* gene, the g.19176G > variant located at the 3′-UTR demonstrates the highest bond and is supposed to enhance the expression of *ITPA* at the post-transcriptional level. A minor ‘A’ allele form of this variant demonstrates a higher level of expression by using a lymphoblastic expression profile (in silico) [4,33]. Eventually, it was speculated that the lower level of expression for the major ‘G’ allele may cause individuals with this allele to be more susceptible to TB infection [4]. This study reveals how the formulation of *ITPA* can play a defensive role in the onset of TB. The human thymus tissue is found to express the highest amount of *ITPA* and the results found after the detailed study of *ITPA* expression in the thymus gland show that it may have a very strong role in the development and outbreak of tuberculosis [4].

### 4.4. Hepatitis C Treatment

Around the globe, approximately 170 million patients are facing hepatitis C, and one of the strongest offshoots after treatment with interferon alpha and ribavirin is haemolytic anaemia [33]. Similarly, ITPase polymorphism (rs1127354) was found to be associated with haemoglobin level in treated Chinese cohorts of hepatitis C [34]. Various studies showed that the variant c.94C > A (p.Pro32Thr) is responsible for delaying the conditions responsible for anaemia. Data show that patients having variants c.94C > A (p.Pro32Thr) or c.124 + 21A > C not only develop the symptoms of anaemia, but also require a low reduction in riboflavin dose [35,36,37]. Nowadays, the *ITPA* activity is used as a probe to detect the development of anaemia in patients taking riboflavin treatment [38]. Based on the above finding, it is advisable to use the ITPase activity or *ITPA* locus in the treatment of hepatitis C using ribavirin [39].

### 4.5. Antiviral Treatment-Driven Anaemia

Apart from that mentioned above, ITPase deficiency was found to have a major impact on the patients treated with antivirals. In general, the patients with a moderate deficiency showed the most protection during the complete course of the antiviral therapy. Under these scenarios, the rate of haemoglobin decline was severe in patients with wild-type ITPase activity. This was found to be highly correlated with a greater deficiency in haemoglobin levels over the full course of therapy.

Overall, the abovementioned shows that *ITPA* mutations have a strong association with treatments for a variety of diseases, including cancer.

## 5. *ITPA* Variants/Mutants: A Disease Range

With the help of different studies, it is now very clear that ITPase plays a very important role in the purine metabolism pathway and *ITPA* variation is responsible for modulated *ITPA* activity/expression, which causes different diseases in different parts of the body [40,41]. Firstly, it was identified by Vanderheiden in patients with the abnormal accumulation of ITP in erythrocytes, and it was suggested that the patients were ITPase deficient [40,42]. However, with more research, in the year 2002, it was shown that there is no harm in the absence of ITPase activity, with zero percent activity from c.94C > A (p.Pro32Thr) homozygous individuals [11,33]. Additionally, a study also showed that ITPase gene variants prevent haemolytic anaemia in treated HCV patients [42]. Today it is understood that the role of this protein is different in all the different tissues of the body. This underscores that there is a very important relationship between the *ITPA* variation and drug-dosage system.

## 6. Final Remarks

In view of the ongoing evidence finding that the phenotypic series of *ITPA* defects ranges from very mild to severe deaths, it was also suggested that therapies will be developed to regulate ITPase activity. The *ITPA* status contributes to several severe diseases, from cardiomyopathy to neural defects and disorders of the immune system, as well as several other *ITPA*-related diseases [4,22,23]. The low-cost development of sequencing techniques, such as NGS and WES, will help to evaluate several other ITPase-related diseases.

## 7. Conclusions

There is a need for further research in order to understand the *ITPA* polymorphism. To date, only seven mutants/variants are known and this has posed a great challenge to clinicians to understand it completely. Several previous studies have indeed provided valuable inputs to increase our understanding of the mechanism involved in the differentiation of canonical and non-canonical purine metabolism and its involvement in disease. Advances in this field, taking the shape of continuous progress, have recently witnessed quantum leaps, but there is still much to study.

## Figures and Tables

**Table 1:** Clinically relevant *ITPA* mutants/variants and their biological impacts.

SNP ID	Variation	Clinical Significance	Biological Significance	Location
rs7270101	SNP	ADR	Poor splicing efficiency	c.124 + 21A > C (g.IVS2 + 21A > C)
rs1127354	SNP	ADR	Reduced expression, stability, catalysis	c.94C > A (p.Pro32Thr)
NA	SNP	Encephalopathy	Altered substrate specificity, poor solubility	c.532C > T (p.Arg178Cys)
rs13830	SNP	Tuberculosis	3′UTR variation, altered mRNA metabolism/translation	g.19176G > A
NA	Nonsense	Encephalopathy	Nonsense RNA-mediated decay	c.452G > A (p.Trp151Stop)
rs863225424	Duplication	Encephalopathy	Frameshift, non-functional protein	c.359_366dupTCAGCACC (p.Gly123Serfs)
NA	Deletion	Encephalopathy	1874 bp deletion, frameshift, non-functional protein	c.264-607_295 + 1267del1906

The overall impacts of *ITPA* mutations or variants are associated with the other diseases and their treatments, such as infantile encephalopathy, cancer chemotherapy, tuberculosis treatment, Hepatitis C treatment, Antiviral treatment etc.

## Data Availability

Not applicable.
